# Supplementary material for: High-performance ionomer-free gas diffusion cathodes with low Pt loading for proton exchange membrane water electrolysis
Source: Commun Mater. 2026 Jan 21;7(1):67. doi: 10.1038/s43246-026-01076-2 (PMC12913019; doi:10.1038/s43246-026-01076-2)
Supplement: Supplementary file 2 — supplementary materials for manuscript COMMSMAT-25-0845B [file 43246_2026_1076_MOESM2_ESM.pdf]

Supplementary Materials for

High-performance ionomer-free gas diffusion cathodes with low Pt loading for proton exchange membrane water electrolysis

Mingliang Chen[1], Peter M. Piechulla[1], Alexandros Mantzanas[1], Mena-Alexander Kräenbring[2], Fatih Özcan[2,3], Doris Segets[2,3], J. Ruud van Ommen[1*]

[1] *Department of Chemical Engineering, Delft University of Technology, 2629 HZ Delft, the Netherlands*

[2] *Institute for Energy and Materials Processes – Particle Science and Technology, Universität Duisburg-Essen, 47057 Duisburg, Germany*

[3] *Center for Nanointegration Duisburg-Essen, Universität Duisburg-Essen, 47057 Duisburg, Germany*

Corresponding author: J. Ruud van Ommen, j.r.vanommen@tudelft.nl

The PDF file includes:

Figs. S1 to S17

Table S1

References

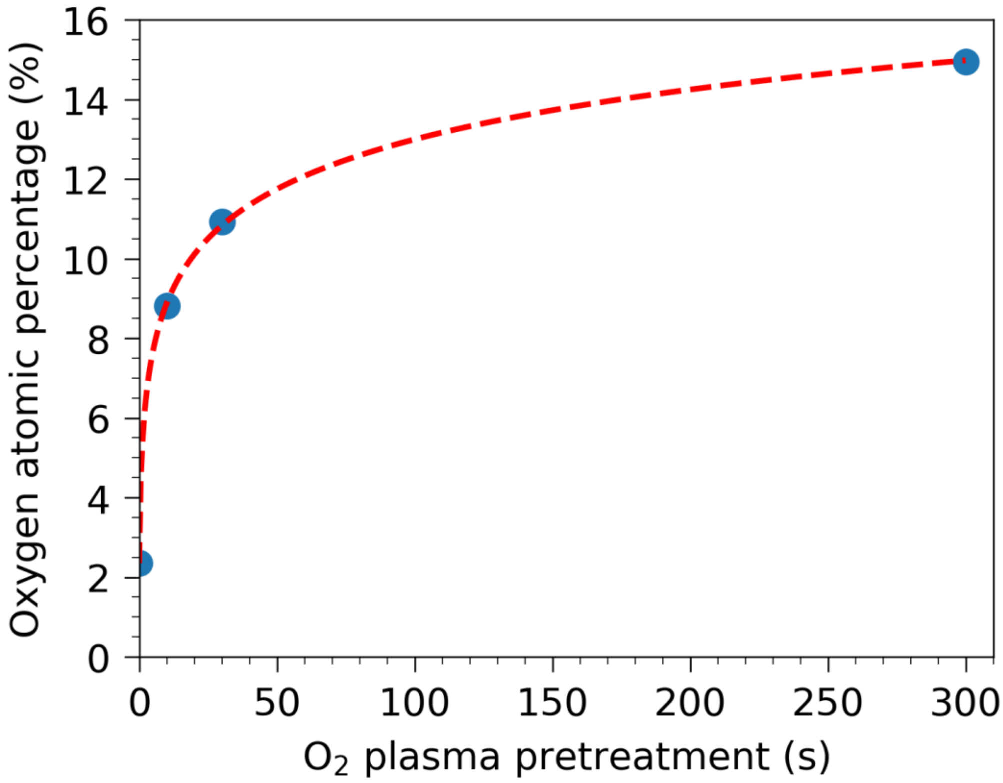

Fig. S1. Effect of O_2 plasma pretreatment on the oxygen concentration on the surface of carbon fibers (H23).

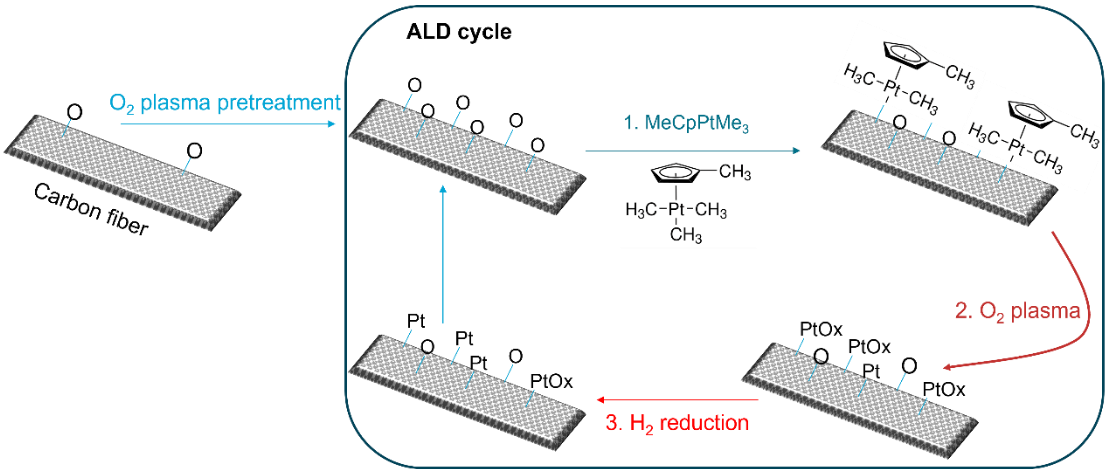

Fig. S2. Schematic of atomic layer deposition (ALD) of Pt on carbon fiber surface. The carbon fiber was pretreated with O_2 plasma prior to ALD.

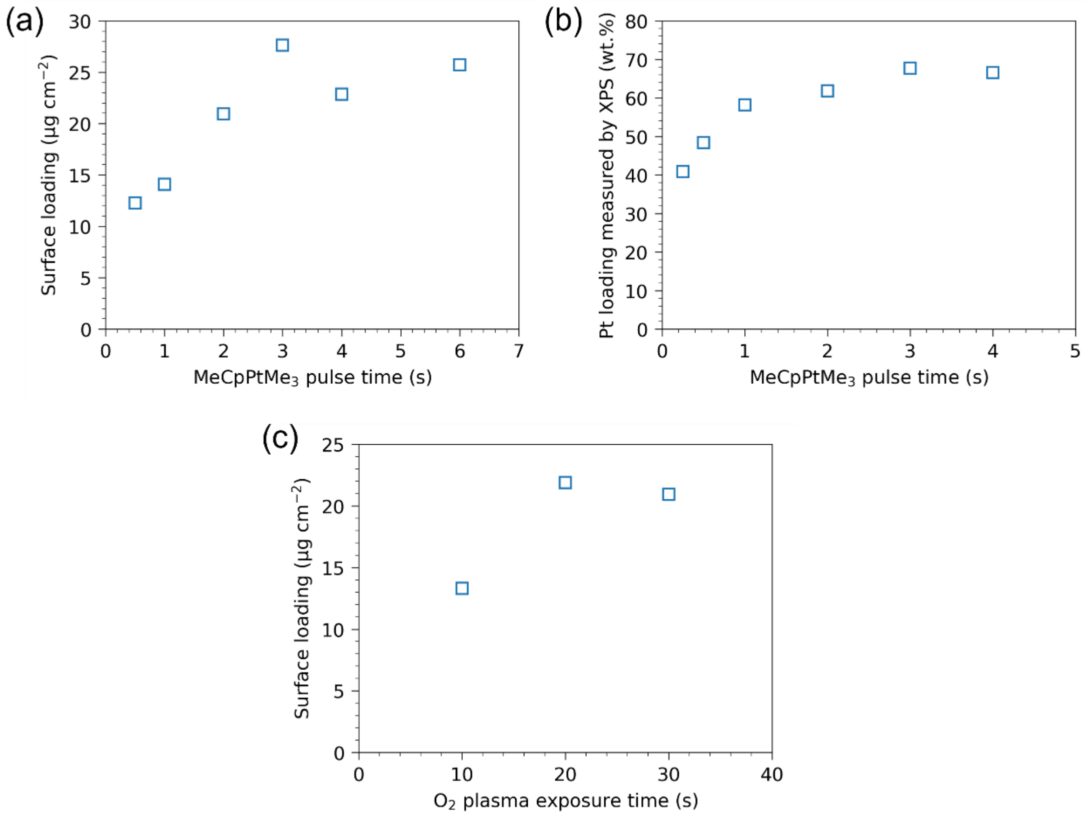

Fig. S3. Effect of $MeCpPtMe_3$ pulse time and O_2 plasma exposure time on geometrical surface loading of Pt on carbon fiber (H23). The loading of Pt in (a) and (c) was measured by inductively coupled plasma mass spectrometry (ICP-MS) and then converted to surface loading, while the loading of Pt on carbon fiber in (b) was measured by X-ray photoemission spectroscopy (XPS).

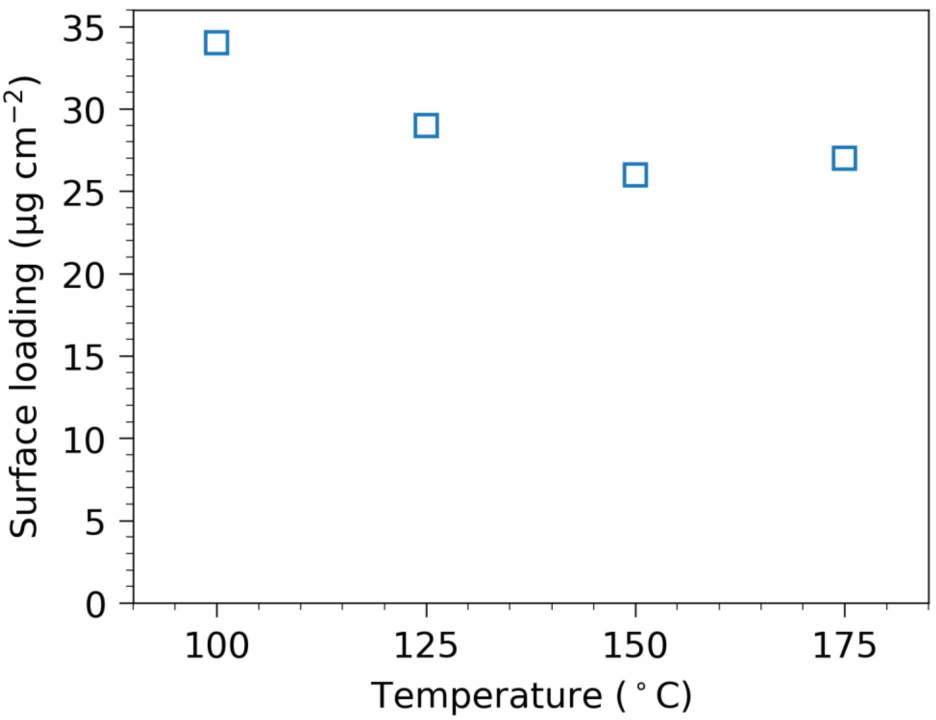

Fig. S4. Effect of ALD reactor temperature on Pt loading on carbon fiber (H23).

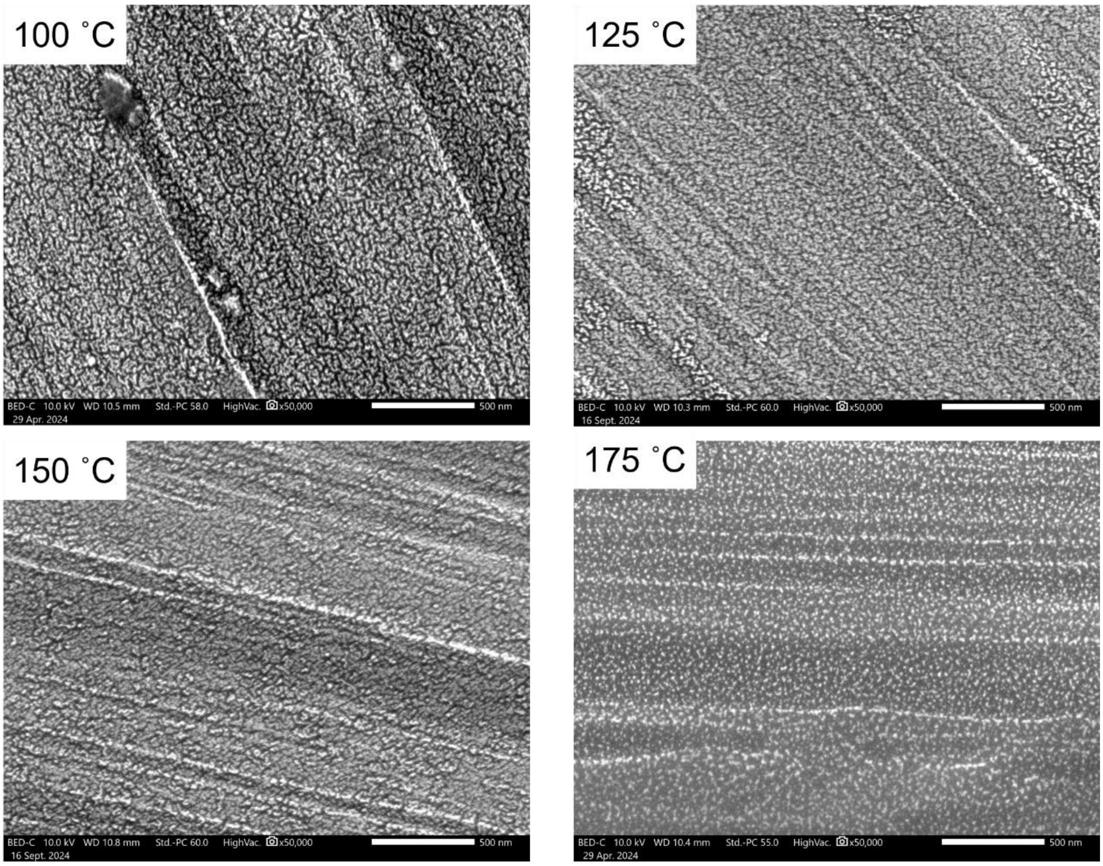

Fig. S5. Surface morphology of carbon fiber (H23) after ALD deposition at various temperatures. The scale bar of the SEM images is 500 nm.

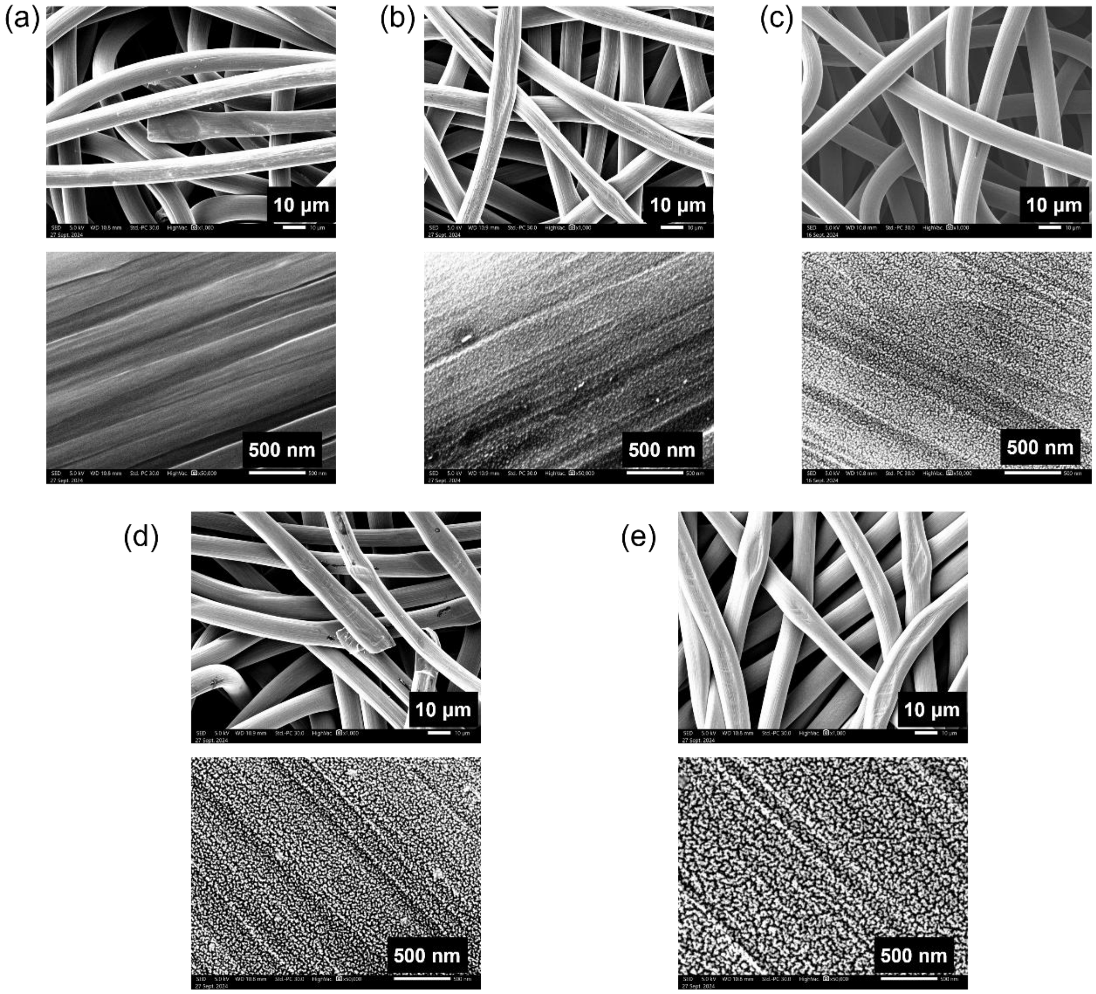

Fig. S6. SEM images of GDL (H23) before and after deposition of Pt using ALD. (a) pristine GDL without deposition, (b) ALD_N15, (c) ALD_N30, (d) ALD_50, (e) ALD_N100.

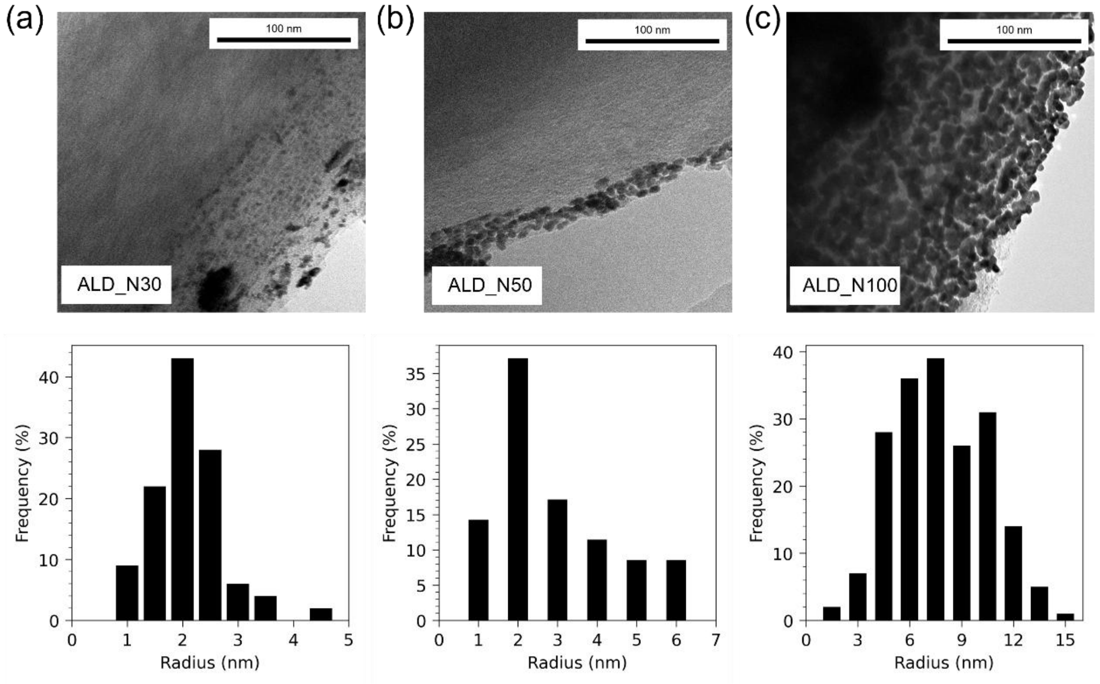

Fig. S7. TEM images and the corresponding particle size distribution of Pt on GDL (H23) after different number of ALD cycles. (a) ALD_N30, (b) ALD_N50, (c) ALD_N100.

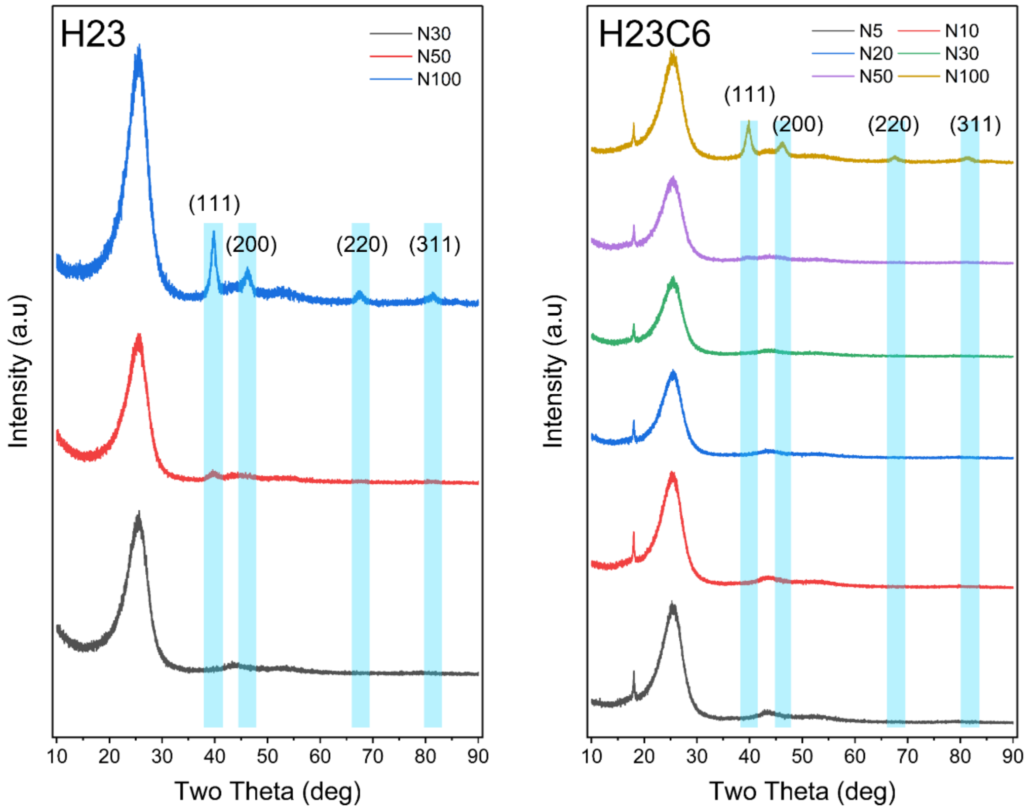

Fig. S8. XRD patterns of H23 and H23C6 after ALD deposition at different number of cycles.

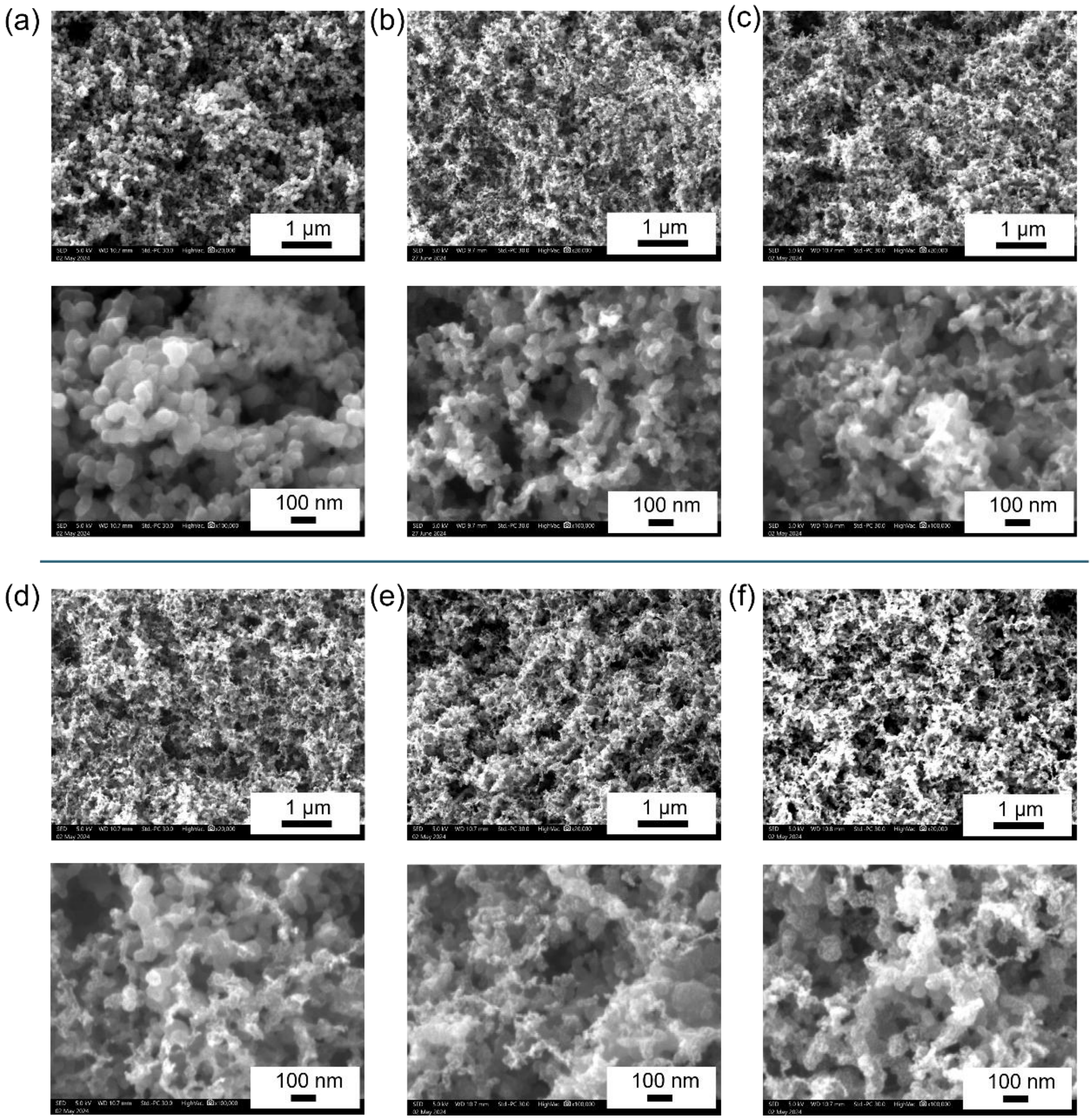

Fig. S9. SEM images of GDL (H23C6) before and after deposition of Pt using ALD at different number of cycles. (a) pristine GDL without deposition, (b) ALD_N5_MPL, (c) ALD_N10_MPL, (d) ALD_N20_MPL, (e) ALD_N30_MPL, (f) ALD_N50_MPL.

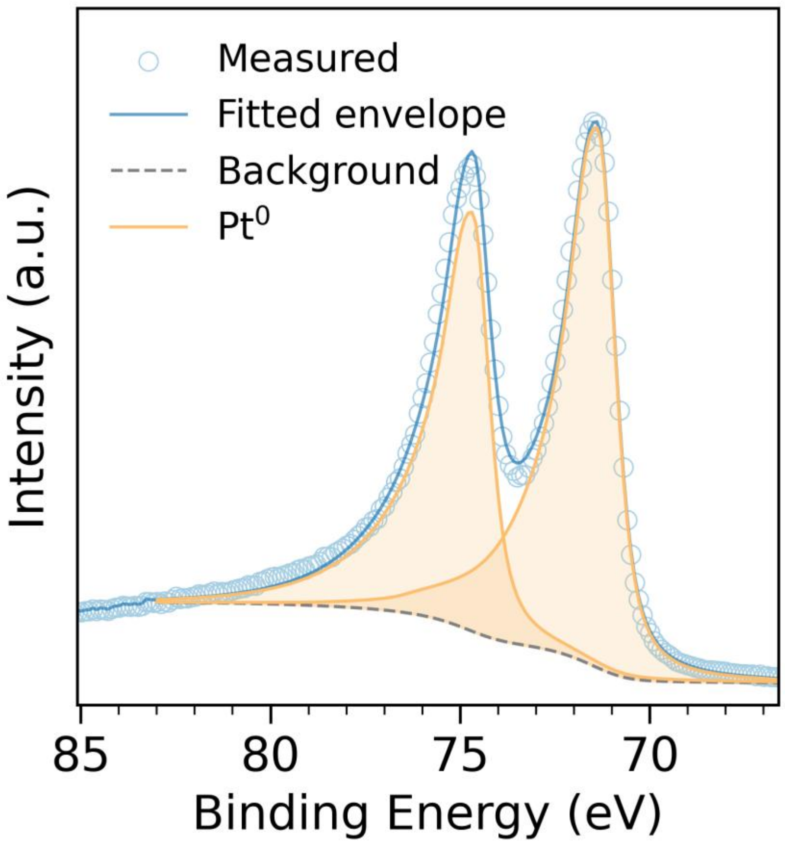

Fig. S10. Pt 4f XPS spectra of ALD_N10_MPL after 200 hours stability test. All Pt oxides were reduced to metallic Pt after operation.

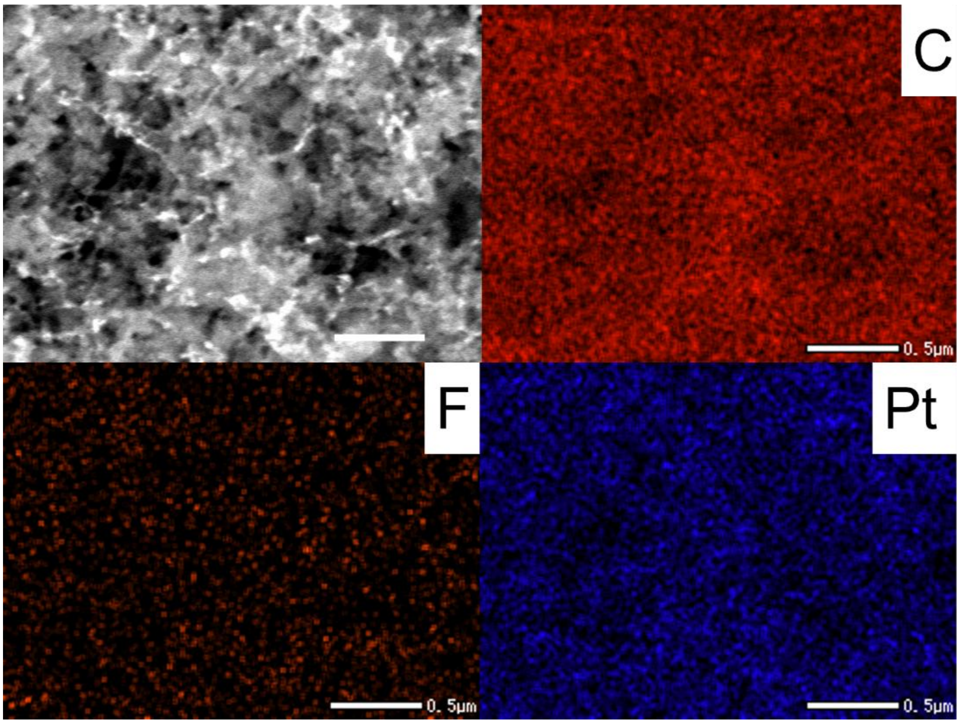

Fig. S11. EDS surface mapping of ALD_N20_MPL. The scale bar is 500 nm.

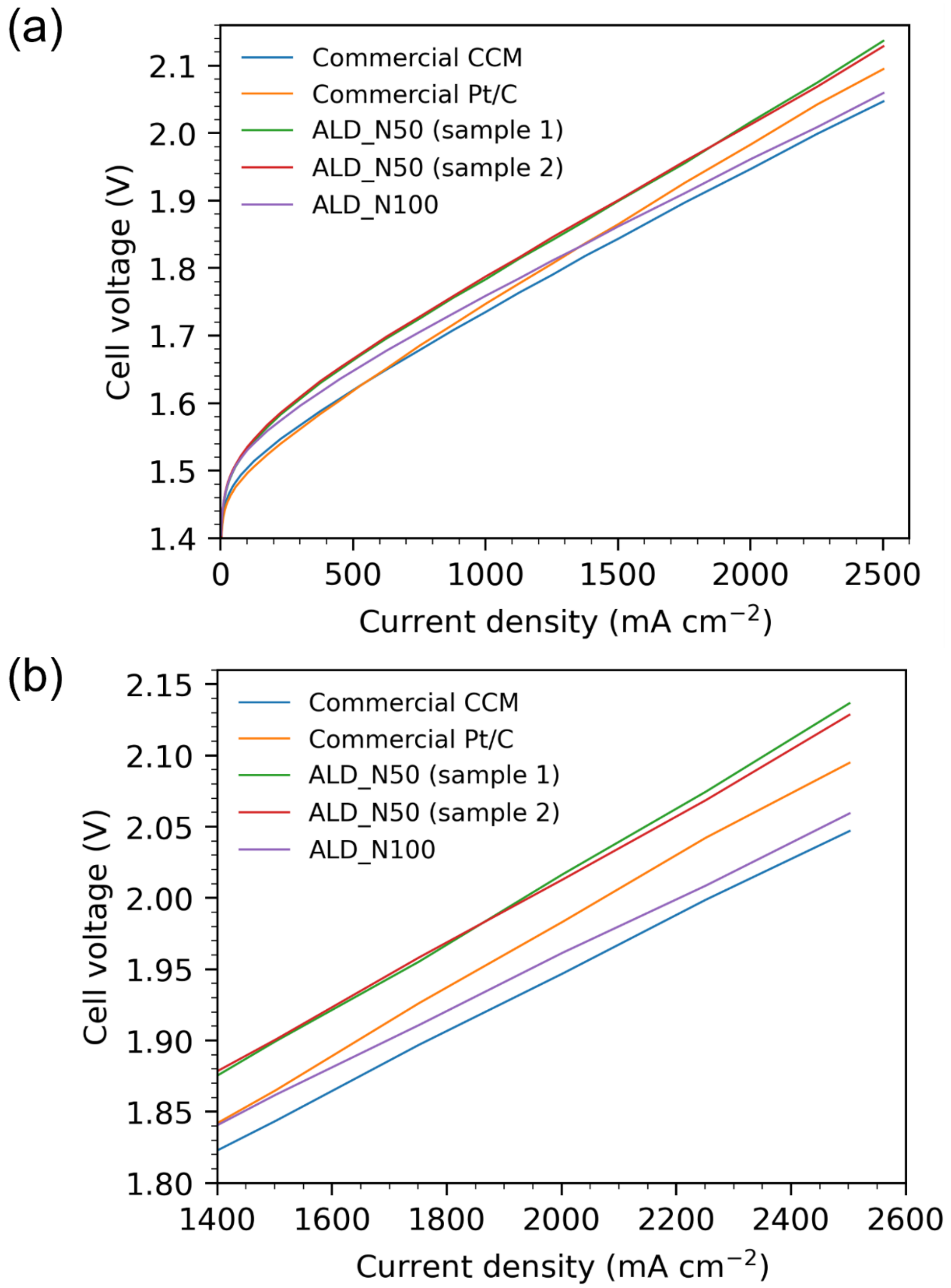

Fig. S12. Comparison of current polarization curves of ALD GDEs on H23 with commercial CCM and spray-coated Pt/C GDE.

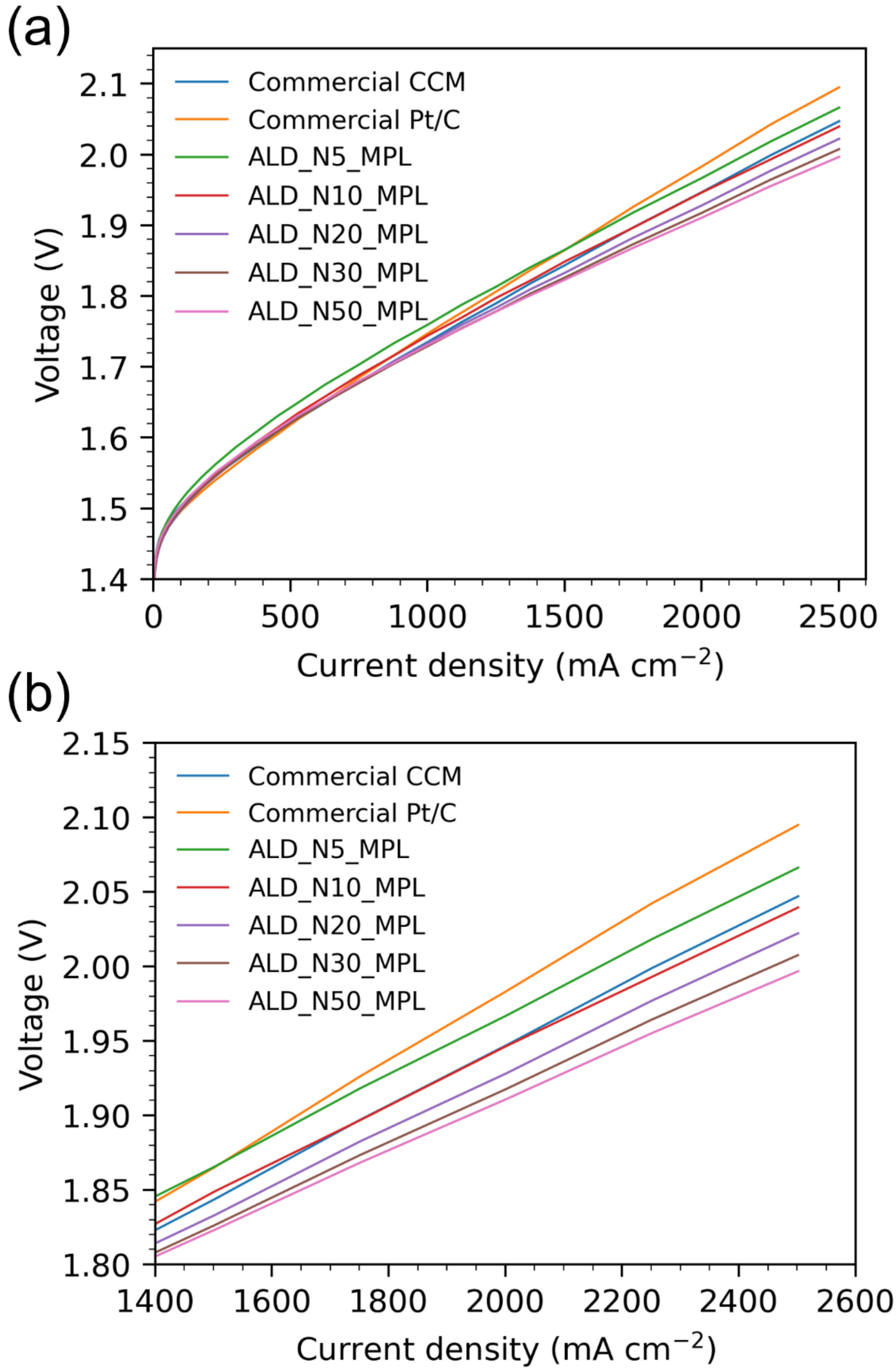

Fig. S13. Comparison of current polarization curves of ALD GDEs on H23C6 with commercial CCM and spray-coated Pt/C GDE.

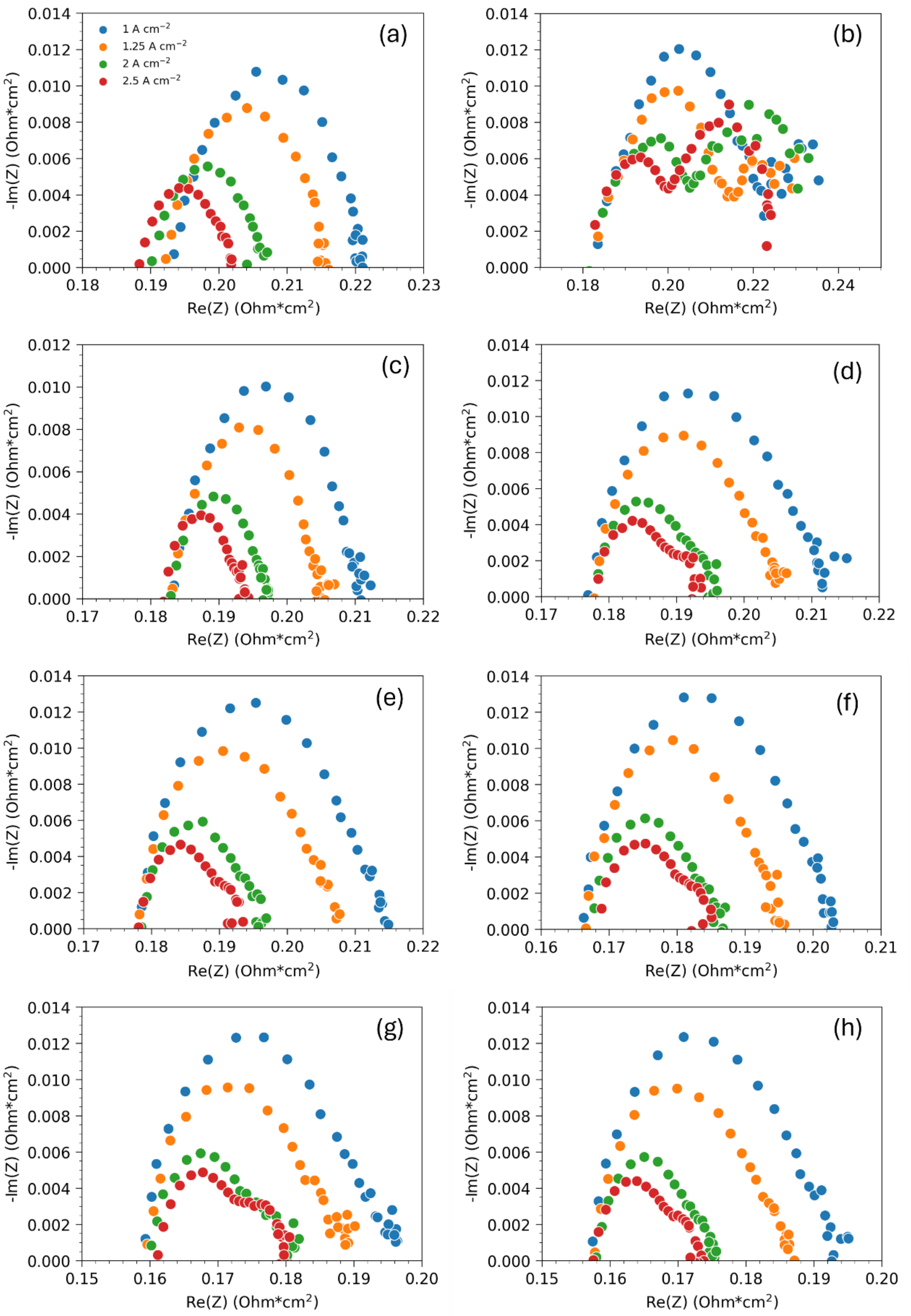

Fig. S14. EIS curves of different MEAs measured at a frequency ranging from 100 kHz to 100 mHz. (a) commercial CCM, (b) commercial Pt/C GDE made by spray coating method, (c) ALD_N100, (d) ALD_N5_MPL, (e) ALD_N10_MPL, (f) ALD_N20_MPL, (g) ALD_N30_MPL, (h) ALD_N50_MPL.

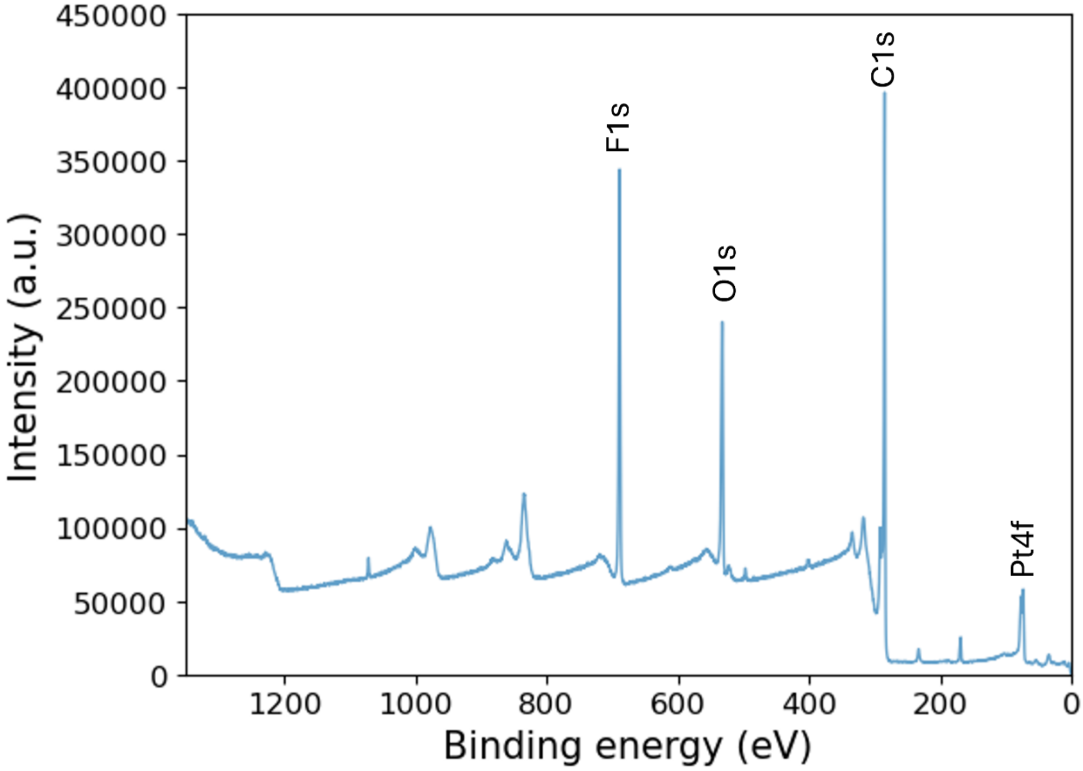

Fig. S15. XPS survey scan of ALD_N5_MPL which demonstrates that the main elements present on the surface are F, O, C and Pt.

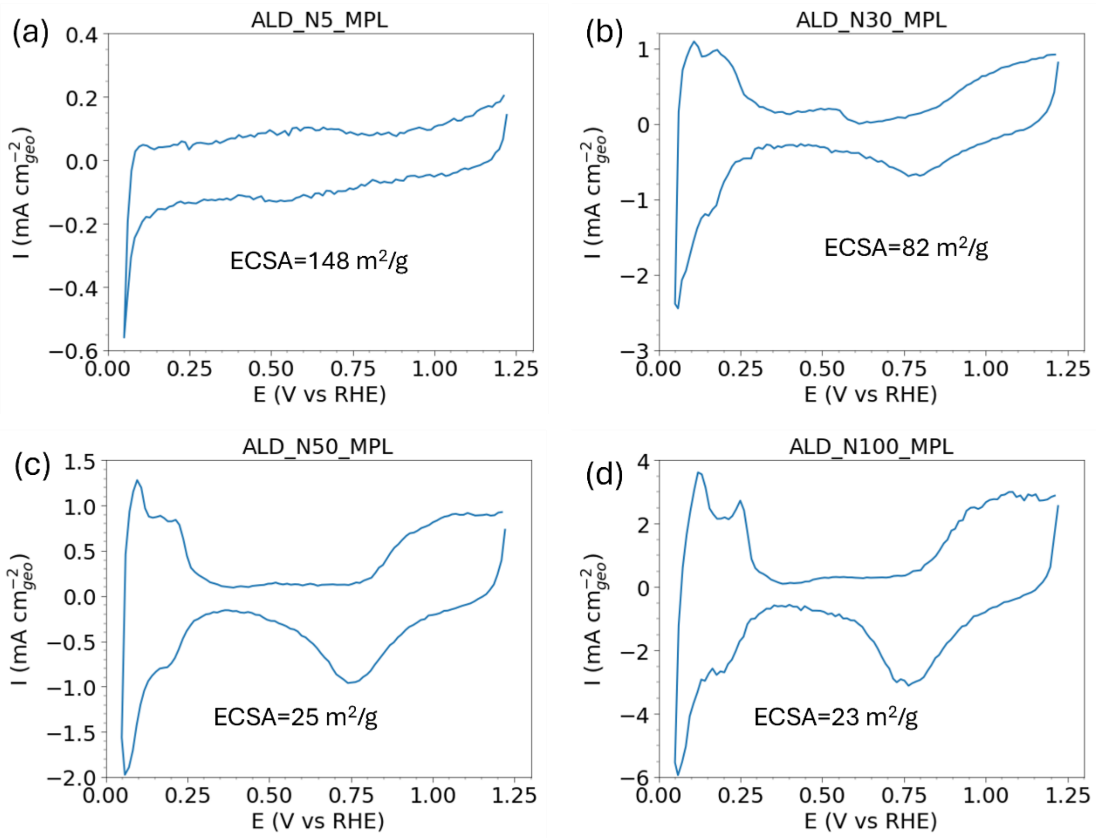

Fig. S16. Electrochemical surface area of ALD GDEs on H23C6. (a) ALD_N5_MPL, (b) ALD_N30_MPL, (c) ALD_N50_MPL, (d) ALD_N100_MPL.

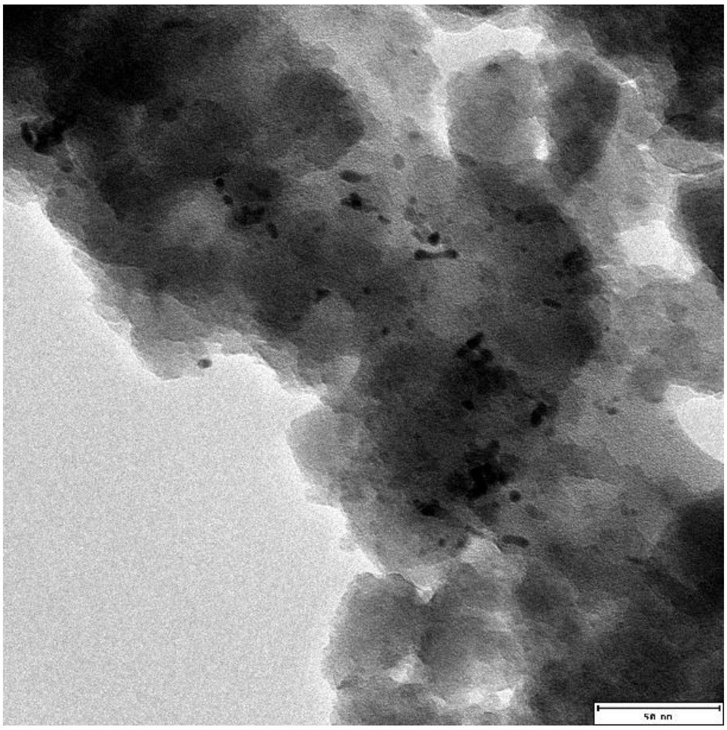

Fig. S17. TEM analysis of the GDE after 25,000-cycle durability test. Due to the cell compression and the durability test at 80 °C, most of Pt particles were likely transferred to the membrane, and only a small number of them remained on the GDE.

Table S1. Comparison of the PEMWE cell performance in this work with those from the state-of-the-art studies in literature.

Anode catalyst	Cathode catalyst	Cathode abbreviation	Anode loading (mg/cm^2)	Cathode loading (mg/cm^2)	Membrane	Tem[a] (˚C)	Voltage (V)	Current (A/cm^2)	Performance[b] (kW/g)	Refs
Ir-black	ALD_N5_ MPL	ALD_N5_MPL	1.4	1.08E-03	Nafion-117	80	1.8	1.19	1.98E+03	This work
Ir-black	ALD_N10_ MPL	ALD_N10_MPL	1.4	5.40E-03	Nafion-117	80	1.8	1.27	4.23E+02	This work
IrO_2	Pt/C	Pt/C	2.5	0.5	~	80	1.8	1.4	5.04E+00	[1]
IrO_2	Pt/C	Pt/C	2.5	0.5	~	80	1.8	1.1	3.96E+00	[1]
IrO_2/TiO_2	Pt/C	Pt/C	2	0.025	Nafion-212	80	1.8	2.75	1.98E+02	[2]
$IrRuO_x$	40%Pt/C	Pt/C	3	0.3	Nafion-115	60	1.8	0.8	4.80E+00	[3]
$IrRuO_x$	sputtered Pt	Pt	3	0.1	Nafion-115	60	1.8	0.9	1.62E+01	[3]
IrO_2	Pt/C	Pt/C	2	1	Nafion-212	80	1.8	1.6	2.88E+00	[4]
IrO_2	Pt/CNF	Pt/CNF	1	0.077	Nafion-115	80	1.8	1.48	3.46E+01	[5]
IrO_2	Pt/CNF	Pt/CNF	1	0.015	Nafion-115	80	1.8	1.35	1.62E+02	[5]
IrO_2	Pt/CNF	Pt/CNF	1	0.009	Nafion-115	80	1.8	1	2.00E+02	[5]
RuO_2	Pt/C	Pt/C	1.4	0.4	~	80	1.8	1.9	8.55E+00	[6]
IrO_2	Alloyed Pt Single-Atom	Pt	2.5	0.02	Nafion-117	80	1.8	1.3	1.17E+02	[7]
IrO_2	40%Pt/C	Pt/C		1	Nafion-117	80	1.8	1.25	2.25E+00	[8]
IrO_2	60% Pt/C	Pt/C	1.2	0.5	Nafion-212	80	1.8	2	7.20E+00	[9]
IrO_2/TiO_2	60% Pt/C	Pt/C	1.4	0.5	Nafion-212	80	1.8	2.4	8.64E+00	[9]
IrO_2@TiO_2	60% Pt/C	Pt/C	1.4	0.5	Nafion-212	80	1.8	3	1.08E+01	[9]
IrO_2	Pt/C	Pt/C	1.5	0.5	Nafion-115	80	1.8	2	7.20E+00	[10]

RuO_2	30%Pd/PN-CNPs	Pd/PN-CNPs	3	0.7	Nafion-115	80	1.8	0.6	1.54E+00	[11]
IrO_2	Pt/C	Pt/C	1.4	0.5	Nafion-117	80	1.8	1.25	4.50E+00	[12]
RuO_2	30%Pd/P-CNPs	Pd/P-CNPs	3	0.7	Nafion-115	80	1.8	0.5	1.29E+00	[13]
RuO_2	30%Pd/PG	Pd/PG	3	0.7	Nafion-115	80	1.8	0.65	1.67E+00	[14]
IrO_2/TiO_2	4.8%Pt/C	Pt/C	1.6	0.025	Nafion-212	80	1.8	4	2.88E+02	[15]
RuO_2	30%Pd/N-CNT	Pd/N-CNT	3	0.7	Nafion-115	80	1.8	0.9	2.31E+00	[16]
RuO_2	46% Pt/C	Pt/C	1	0.2	Nafion-117	80	1.8	1.5	1.35E+01	[17]
$Ru_{0.9}Ir_{0.1}O_2$	46% Pt/C	Pt/C	1	0.2	Nafion-117	80	1.8	1.2	1.08E+01	[17]
$Ru_{0.7}Ir_{0.3}O_2$	46% Pt/C	Pt/C	1.6	0.2	Nafion-117	80	1.8	1.25	1.13E+01	[17]
IrO_2	46% Pt/C	Pt/C	1.2	0.2	Nafion-117	80	1.8	1	9.00E+00	[17]
Ir-black	40% Pt/GNF	Pt/GNF	2	0.8	Nafion-115	90	1.8	1.7	3.83E+00	[18]
Ir-black	40% Pt/XC72	Pt/C	2	0.8	Nafion-115	90	1.8	1.32	2.97E+00	[18]
Ir-black	Pt-black	Pt	2	0.8	Nafion-117	90	1.8	1.3	2.93E+00	[19]
RuO_2	30% Pt/C	Pt/C	1.5	0.5	Nafion-1035	80	1.8	1.9	6.84E+00	[20]
IrO_2	30% Pt/C	Pt/C	1.5	0.5	Nafion-1035	80	1.8	1.6	5.76E+00	[20]
Ir-black	Pt40/XC-72	Pt/C	2.4	0.7	Nafion-115	90	1.8	1.65	4.24E+00	[21]
Ir-black	Pd40/XC-72	Pt/C	2.4	0.7	Nafion-115	90	1.8	1.5	3.86E+00	[21]

CNF: carbon nanofiber, PN-CNP: phosphorus–nitrogen dual-doped carbon nanoparticles, P-CNP: phosphorous-doped carbon nanoparticles, N-CNT: Nitrogen-doped carbon nanotube, PG: phosphorus-doped graphene, GNF: graphitic nanofiber, a = Temperature, ~ = not mentioned, b: the calculation is

based on the cathode Pt loading.

References

1. C. Cesar Weber *et al.*, *EES Catalysis* **2**, 585-602 (2024).
2. Z. Zhang, A. Baudy, A. Testino, L. Gubler, *ACS Appl. Mater. Interfaces* **16**, 23265–23277 (2024).
3. A. Villamayor, A. Alba, L. V. Barrio, S. Rojas, E. Gutierrez-Berasategui, *Coatings* **14**, 868 (2024).
4. Z. Xie *et al.*, *Applied Catalysis B: Environmental* **341**, 123298 (2024).
5. J. Song *et al.*, *ACS Sustainable Chem. Eng.* **11**, 16258-16266 (2023).
6. A. Piñeiro García, D. Perivoliotis, X. Wu, E. Gracia-Espino, *ACS Sustainable Chem. Eng.* **11**, 7641-7654 (2023).
7. H. Gao *et al.*, *Advanced Functional Materials* **33**, 2214795 (2023).
8. L. Shen *et al.*, *Journal of Power Sources* **538**, 231557 (2022).
9. C. V. Pham *et al.*, *Applied Catalysis B: Environmental* **269**, 118762 (2020).
10. P. K. R. Holzapfel *et al.*, *Small* **16**, 2003161 (2020).
11. S. Shiva Kumar, S. U. B. Ramakrishna, K. Naga Mahesh, B. Rama Devi, V. Himabindu, *Ionics* **25**, 2615-2625 (2019).
12. M. Bühler *et al.*, *J. Mater. Chem. A* **7**, 26984-26995 (2019).
13. S. Shiva Kumar, S. U. B. Ramakrishna, B. Rama Devi, V. Himabindu, *Ionics* **24**, 3113-3121 (2018).
14. S. Shiva Kumar, S. Ramakrishna, B. Rama Devi, V. Himabindu, *International Journal of Green Energy* **15**, 558-567 (2018).
15. M. Bernt, A. Siebel, H. A. Gasteiger, *J. Electrochem. Soc.* **165**, F305 (2018).
16. S. U. B. Ramakrishna, D. Srinivasulu Reddy, S. Shiva Kumar, V. Himabindu, *Int. J. Hydrog. Energy* **41**, 20447-20454 (2016).
17. T. Audichon *et al.*, *Int. J. Hydrog. Energy* **39**, 16785-16796 (2014).
18. S. A. Grigoriev, M. S. Mamat, K. A. Dzhus, G. S. Walker, P. Millet, *Int. J. Hydrog. Energy* **36**, 4143-4147 (2011).
19. S. A. Grigoriev *et al.*, *Int. J. Hydrog. Energy* **34**, 5986-5991 (2009).
20. J. Cheng, H. Zhang, G. Chen, Y. Zhang, *Electrochimica Acta* **54**, 6250-6256 (2009).
21. S. A. Grigoriev, P. Millet, V. N. Fateev, *Journal of Power Sources* **177**, 281-285 (2008).
