# Supplementary material for: High-performance ionomer-free gas diffusion cathodes with low Pt loading for proton exchange membrane water electrolysis
Source: Commun Mater. 2026 Jan 21;7(1):67. doi: 10.1038/s43246-026-01076-2 (PMC12913019; doi:10.1038/s43246-026-01076-2)
Supplement: Supplementary file 3 — Description of Additional Supplementary File [file 43246_2026_1076_MOESM3_ESM.pdf]

Supplementary Data 1: Source data for Figure 2 [Characterization of Pt gas diffusion electrode].

Supplementary Data 2: Source data for Figure 3 [Three different types of MEAs are compared in terms of current polarization curves].

Supplementary Data 3: Source data for Figure 4 [Catalyst layer utilization and performance comparison with those from literature].

Supplementary Data 4: Source data for Figure 5 [Long-term stability].

Supplementary Data 5: Source data for Figure 6 [Durability test in dynamic operation].
